# Multivariate Analysis As a Support for Diagnostic Flowcharts in Allergic Bronchopulmonary Aspergillosis: A Proof-of-Concept Study

**DOI:** 10.3389/fimmu.2017.01019

**Published:** 2017-08-22

**Authors:** Joana Vitte, Stéphane Ranque, Ania Carsin, Carine Gomez, Thomas Romain, Carole Cassagne, Marion Gouitaa, Mélisande Baravalle-Einaudi, Nathalie Stremler-Le Bel, Martine Reynaud-Gaubert, Jean-Christophe Dubus, Jean-Louis Mège, Jean Gaudart

**Affiliations:** ^1^Aix-Marseille Univ, APHM Assistance Publique Hôpitaux de Marseille, Hôpital de La Conception, Laboratoire d’Immunologie, Marseille, France; ^2^Aix-Marseille Univ, UMR INSERM 1067 CNRS 7333, Marseille, France; ^3^Aix-Marseille Univ, APHM Assistance Publique Hôpitaux de Marseille, Hôpital Timone, Laboratoire de Parasitologie, Marseille, France; ^4^Aix-Marseille Univ, URMITE, UMR 63, CNRS 7278, INSERM U1095, IRD 198, Marseille, France; ^5^Aix-Marseille Univ, APHM Assistance Publique Hôpitaux de Marseille, Hôpital Timone Enfants, Pneumo-pédiatrie, Centre de Ressources et de Compétences en Mucoviscidose, Marseille, France; ^6^Aix-Marseille Univ, APHM Assistance Publique Hôpitaux de Marseille, Hôpital Nord, Centre de Ressources et de Compétences en Mucoviscidose, Marseille, France; ^7^Aix-Marseille Univ, APHM Assistance Publique Hôpitaux de Marseille, Hôpital Nord, Service de Pneumologie, Marseille, France; ^8^Aix Marseille Univ, IRD, INSERM, SESSTIM UMR 912, Faculté de Médecine campus Timone, Marseille, France

**Keywords:** allergic bronchopulmonary aspergillosis, *Aspergillus fumigatus*, immunoglobulin, molecular allergens, multivariate analysis

## Abstract

Molecular-based allergy diagnosis yields multiple biomarker datasets. The classical diagnostic score for allergic bronchopulmonary aspergillosis (ABPA), a severe disease usually occurring in asthmatic patients and people with cystic fibrosis, comprises succinct immunological criteria formulated in 1977: total IgE, anti-*Aspergillus fumigatus* (*Af*) IgE, anti-*Af* “precipitins,” and anti-*Af* IgG. Progress achieved over the last four decades led to multiple IgE and IgG(4) *Af* biomarkers available with quantitative, standardized, molecular-level reports. These newly available biomarkers have not been included in the current diagnostic criteria, either individually or in algorithms, despite persistent underdiagnosis of ABPA. Large numbers of individual biomarkers may hinder their use in clinical practice. Conversely, multivariate analysis using new tools may bring about a better chance of less diagnostic mistakes. We report here a proof-of-concept work consisting of a three-step multivariate analysis of *Af* IgE, IgG, and IgG4 biomarkers through a combination of principal component analysis, hierarchical ascendant classification, and classification and regression tree multivariate analysis. The resulting diagnostic algorithms might show the way for novel criteria and improved diagnostic efficiency in *Af*-sensitized patients at risk for ABPA.

## Introduction

*Aspergillus fumigatus* (*Af*) is a ubiquitous airborne fungus clinically relevant in asthmatic, cystic fibrosis (CF), and immunosuppressed patients. *Af*–human host interaction spans asymptomatic immunization with detectable immunoglobulin G to *Af*, sensitization with detectable IgE to *Af*, through severe pulmonary or systemic diseases such as allergic bronchopulmonary aspergillosis (ABPA), chronic pulmonary aspergillosis, invasive aspergillosis, and chronic lung allograft disease (1).

Diagnosis of *Af*-related disease relies on a body of clinical, radiological, immunological and mycological evidence. Among *Af*-related diseases in humans, ABPA or Hinson and Pepys’ disease (2, 3) is the most frequent, preferentially targeting CF and asthmatic patients. The estimated prevalence of ABPA is 8–10% (range 1–25%) among CF patients and 1–2% among asthmatic ones (4–6). Lung transplantation does not completely prevent ABPA (7). ABPA diagnosis is particularly arduous in CF patients, as they may experience cough, bronchospasm, intercurrent infections, radiological abnormalities, *Af* colonization, and/or sensitization without true ABPA (4). In addition, mere colonization or sensitization by *Af* has been shown to contribute to the deterioration of respiratory function, in pediatric and adult CF patients (6, 8–11). A chronic disease made up of a succession of flare up and remission, ABPA has a major social burden impact, threatening people with asthma, 334 million people worldwide (12) and CF, around 70,000 people (13).

The diagnostic score for ABPA, established in 1977 and updated in 2013, includes succinct immunological criteria: total IgE, anti-*Af* IgE, anti-*Af* “precipitins,” and anti-*Af* IgG (14). Progress achieved over the last four decades in the understanding and workup of *Af*—immune response cross talk led to the identification of multiple IgE and IgG biomarkers, with quantitative, standardized, molecular-level reports (15–17). Yet, the newly available biomarkers are not included in ABPA diagnostic criteria, either individually or in algorithms, despite favorable reports (1) and persisting underdiagnosis of ABPA (4). This prompted us to apply multivariate statistical analysis to *Af*-related immune biomarkers in search for a discriminant yet doctor-friendly diagnostic tool. We present here a proof-of-concept work on the potential benefit of multivariate algorithms for the diagnosis of ABPA.

## Materials and Methods

The statistical analysis was performed retrospectively using flowcharts and laboratory data from 39 ABPA-free CF patients with detectable sIgE to *Af* extract (*Af*-CF group) and 10 ABPA patients (7 asthmatics, 3 CF; 2 children) from the Adult and Pediatric Regional Centers for Cystic Fibrosis (RCCF) and the Pulmonology Departments of Marseille, Southern France. Patient demography and detailed sIgE and sIgG4 data were previously described (17). Data from one ABPA patient (CF background, 12-year-old male) diagnosed with ABPA in July 2016 were included.

sIgE, sIgG, and sIgG4 for *Af* extract and recombinant allergens Asp f 1, Asp f 2, Asp f 3, Asp f 4, and Asp f 6 were measured with Thermo Fisher ImmunoCAP (Uppsala, Sweden). The detection thresholds were 0.01 mg_A_/L for sIgG and sIgG4 and 0.11 kU_A_/L for sIgE. In 44 of these 49 patients, determination of IgG to *Af* was also performed with an ELISA kit (Orgentec, Trappes, France), and *Af* precipitins were evaluated by immunoelectrophoresis (Sebia, Evry, France). ABPA patients were assayed at the initial diagnosis of ABPA or during a subsequent flare. The diagnosis of ABPA relied on (i) acute or subacute pulmonary function deterioration in patients with asthma or CF; (ii) total IgE levels of 500 kIU/L or higher; (iii) elevated levels of specific IgE and either sIgG or precipitins to *Af*; and (iv) chest or computed tomographic pulmonary infiltrates, with pathognomonic high attenuation mucous plugging. Therapeutic unresponsiveness to antibiotics, followed by resolution under corticosteroid treatment, was an additional criterion. Skin prick test reactivity for *Af* was not assessed because of discontinued availability of fungal *Af* extracts.

### Ethics Statement

Recombinant *Af* allergen sIgE and sIgG (4) determination was part of the regular medical care since their commercial release. Patients received written laboratory workup reports. The study was based on a retrospective, non-interventional review of medical charts and laboratory results. According to the French law (18, 19), ethical approval and patient consent were not necessary for this type of study, while patients were informed and retained the right to oppose the use of their anonymized medical data for research purposes.

## Statistical Analysis

First, we performed a hierarchical classification on principal components, as previously described (20, 21). All the variables (biomarker measures) were included in this analysis and were equally weighted. A principal component analysis (PCA) was the first preprocessing step to explore the biomarker on the mixed data set, which takes into consideration relationships between biomarkers. Then, the coordinates of each variable in the first 20 principal components, which summarize 95% of the information, were used to perform a hierarchical ascendant classification (HAC). This method provides classes according to the immunological profile using an objective non-supervised classification technique that allows classifying independently from diagnosis. Furthermore, using the first 20 principal components is a way of denoising the data, thus yielding a more robust classification (21).

Next, we performed a classification and regression tree (CART) multivariate analysis. CART is a supervised, non-parametric and non-linear regressive approach (22), which classified the patients according to the outcome binary variable ABPA or *Af*-CF. Among all covariates, CART analyzed each possible threshold to split the sample in two opposite homogeneous groups. This process was recursively repeated until an optimal criterion was reached. The process enabled a tree to be built in which the terminal classes were groups with common biomarker findings. Statistical analysis was performed using R2.13.0 (R Foundation for Statistical Computing; http://cran.r-project.org/).

## Results

**Figure 1:**
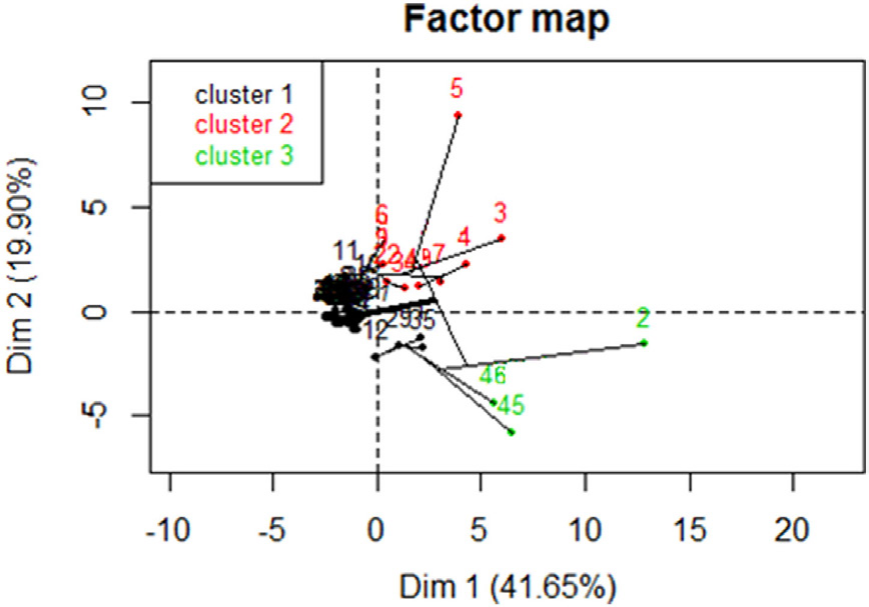
Hierarchical ascendant classification on principal component analysis (PCA) of the study sample. *X*- and *Y*-axes are the two first dimensions issued from the PCA. Patients are denoted 1–10 for the allergic bronchopulmonary aspergillosis (ABPA) group and 11–49 for the *Aspergillus fumigatus* (*Af*)-cystic fibrosis (CF) group. *Af*-sensitized patients without ABPA (*Af*-CF) are mostly found in the homogenous cluster 1 (34/39), while ABPA patients are mostly found in cluster 2 (6/10).

The *PCA-HAC* approach identified three clusters within the population, with a homogenous cluster 1 comprising 34/39 *Af*-CF patients, cluster 2 comprising 6/10 ABPA patients, and cluster 3 including both ABPA and *Af*-CF patients (Figure 1).

Classification and regression tree multivariate analysis method applied to current biomarkers of ABPA (total IgE, sIgE to *Af*, ELISA IgG to *Af*, and *Af* precipitins) resulted in a classification based on three parameters only: total IgE, ELISA IgG, and sIgE. Together, these three results allowed correct classification of 38/39 *Af*-CF patients and 3/10 ABPA patients. The remaining seven ABPA patients were correctly classified with a probability of 88% (Figure S1 in Supplementary Material).

Classification and regression tree was sequentially performed on datasets of either sIgE, IgG or IgG4 responses to *Af* molecules Asp f 1, Asp f 2, Asp f 3, Asp f 4, and Asp f 6. The molecular sIgE dataset allowed proper classification of 35/39 *Af*-CF patients and 8/10 ABPA, the latter with an 89% probability (Figure S2 in Supplementary Material).

Datasets of either IgG or IgG4 responses to molecules Asp f 1, Asp f 2, Asp f 3, Asp f 4, and Asp f 6 resulted in 70–80% of incorrect classification of ABPA patients (not shown), in line with previous IgG4 results (17).

**Figure 2:**
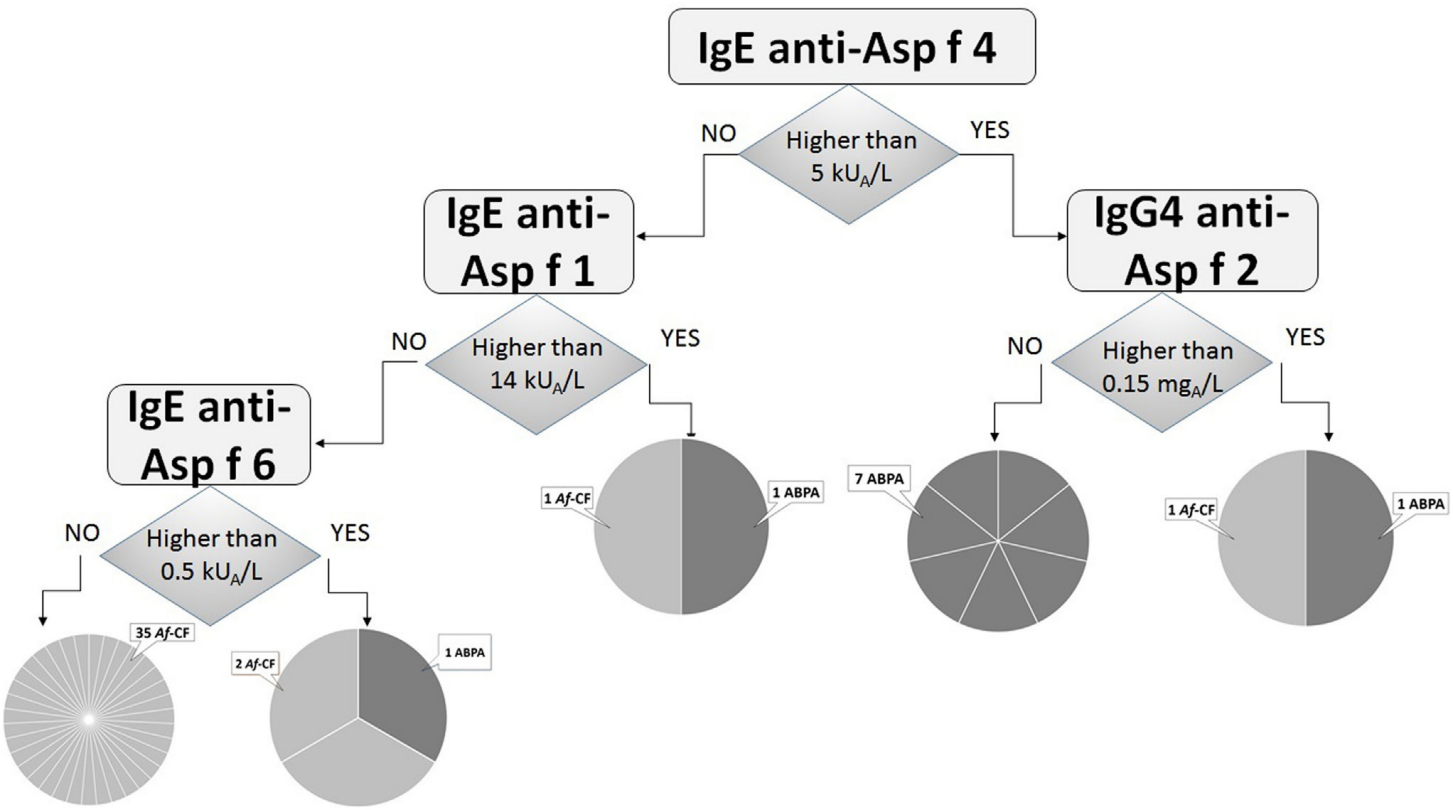
Classification tree using analysis of sIgE, sIgG, and sIgG4 responses to Asp f 1, Asp f 2, Asp f 3, Asp f 4, and Asp f 6 molecules. The diagnostic algorithm retains IgE to Asp f 4, Asp f 1, Asp f 6, and IgG4 to Asp f 2, yielding 1 *Aspergillus fumigatus* (*Af*)-cystic fibrosis (CF)-only groups counting 35/39 patients, 1 allergic bronchopulmonary aspergillosis (ABPA)-only group of 7 patients, and 3 mixed groups of undetermined clinical significance where other criteria are needed. Overall, 7/10 ABPA patients and 35/39 *Af*-CF patients are clearly identified.

Finally, the classification tree built using the three molecular datasets together (sIgE, sIgG, and sIgG4) performed better, and adequately classified 35/39 *Af*-CF and 7/10 ABPA patients. The number of equivocally classified *Af-*CF or ABPA patients was reduced to 4 and 3, respectively (Figure 2).

## Discussion

The present study shows that multivariate analysis approaches can be applied to the analysis of complex *Af*-induced immune response patterns and hold promise for improving diagnostic discrimination between ABPA and ABPA-free patients.

While the multivariate analysis of the current diagnostic criteria (total IgE, sIgE to *Af*, ELISA IgG to *Af*, and *Af* precipitins) correctly classified 38/39 of *Af*-CF patients, only 3/10 ABPA patients were correctly classified (Figure S1 in Supplementary Material). This result suggests that current criteria perform well at identifying ABPA-free patients and excluding ABPA diagnosis but are not optimally efficient for ABPA diagnosis. Shifting from sIgE against *Af* extracts to their molecular counterparts improved ABPA identification. Indeed, the best performance for identifying ABPA cases (8/10 correctly classified patients, Figure S2 in Supplementary Material) was obtained with multivariate analysis of molecular sIgE responses alone, a finding that supports the prominent pathophysiological and diagnostic significance of sIgE responses in ABPA.

The best compromise for identifying both *Af*-CF and ABPA patients was obtained with multivariate analysis of sIgE, IgG, and IgG4 against molecular Asp f 1, Asp f 2, Asp f 3, Asp f 4, and Asp f 6. The algorithm retained only four parameters (sIgE to Asp f 4, Asp f 1, and Asp f 6; IgG4 to Asp f 2) and correctly classified 35/39 *Af*-CF and 7/10 ABPA patients (Figure 2). This result is in line with previous reports on Asp f 4, Asp f 6, and Asp f 1 as ABPA biomarkers [reviewed in Ref. (1)].

In terms of overall diagnostic performance, multivariate analysis still needs improvement. One clue may come from the PCA results, which show that most *Af*-CF cases cluster together (34/39, cluster 1), but ABPA cases only partially do so (6/10, cluster 2, Figure 1). Biological heterogeneity resulting in a mixed *Af*-CF and ABPA cluster 3 needs further work. Differences might be underlain by sIgE or IgG (4) responses to further *Af* molecules currently not available. Conversely, it is likely that genetic, clinical, or radiological features not considered in our study may contribute to *Af*-CF and ABPA heterogeneity. Finally, increasing the size of the study population should improve the power of statistical analysis and yield more performant diagnostic flowcharts.

Taken together, our results add a diagnostic perspective to recent reports of multivariate analysis in allergic patients as a basis for personalized medicine (23). Further confirmation on large-scale populations and detailed analysis of variables are necessary. However, we believe that multivariate analysis of the complex *Af*-induced immune responses will pave the way for the discovery of clinically efficient diagnostic biomarkers and novel ABPA diagnostic algorithms.

## Ethics Statement

Recombinant *Af* allergen sIgE and sIgG (4) determination was part of the regular medical care since their commercial release. Patients received written laboratory workup reports. The study was based on a retrospective, non-interventional review of medical charts and laboratory results. According to the French law (18, 19), ethical approval and patient consent were not necessary for this type of study, while patients were informed and retained the right to oppose the use of their anonymized medical data for research purposes.

## Author Contributions

JV, SR, and JG designed the research. AC, CG, MG, NB, MB-E, MR-G, and J-CD reviewed and collected clinical data. SR and CC interpreted and collected mycological data. TR and JV interpreted and collected immunological data. JG performed statistical analysis. JV, SR, J-CD, MR-G, J-LM, and JG wrote the manuscript. All the authors read and approved the final version of the manuscript.

## Conflict of Interest Statement

The authors declare that the research was conducted in the absence of any commercial or financial relationships that could be construed as a potential conflict of interest.

## Supplementary Material

Figure S1 Classification tree using the classical laboratory measures comprised in the current diagnostic score of allergic bronchopulmonary aspergillosis (ABPA) (redrawn). The diagnostic algorithm retains total IgE, ELISA IgG, and specific IgE, yielding 2 *Aspergillus fumigatus* (*Af*)-cystic fibrosis (CF)-only groups counting 38/39 patients, 1 ABPA-only group of 3 patients, and 1 mixed group of 8 patients with an 88% probability of having ABPA. Overall, 38/39 *Af*-CF patients, but only 3/10 ABPA patients are clearly identified.

Figure S2 Classification tree analyzing sIgE responses to Asp f 1, Asp f 2, Asp f 3, Asp f 4, and Asp f 6 molecules. The resulting diagnostic algorithm retains IgE to Asp f 4, Asp f 1, and Asp f 6, yielding 1 *Aspergillus fumigatus* (*Af*)-cystic fibrosis (CF)-only group counting 35/39 patients, 1 mixed group of 9 patients with an 89% probability of having allergic bronchopulmonary aspergillosis (ABPA), and 2 mixed groups of undetermined clinical significance where other criteria are needed. Overall, 35/39 *Af*-CF patients, but no ABPA patients are clearly identified.

## Abbreviations

ABPA, allergic bronchopulmonary aspergillosis; *Af, Aspergillus fumigatus*; CART, classification and regression tree; CF, cystic fibrosis; HAC, hierarchical ascendant classification; Ig, immunoglobulin; PCA, principal component analysis; s, specific.
