# Supplementary figures and images for: Multivariate Analysis As a Support for Diagnostic Flowcharts in Allergic Bronchopulmonary Aspergillosis: A Proof-of-Concept Study
Source: Front Immunol. 2017 Aug 22;8:1019. doi: 10.3389/fimmu.2017.01019 (PMC5572279; doi:10.3389/fimmu.2017.01019)

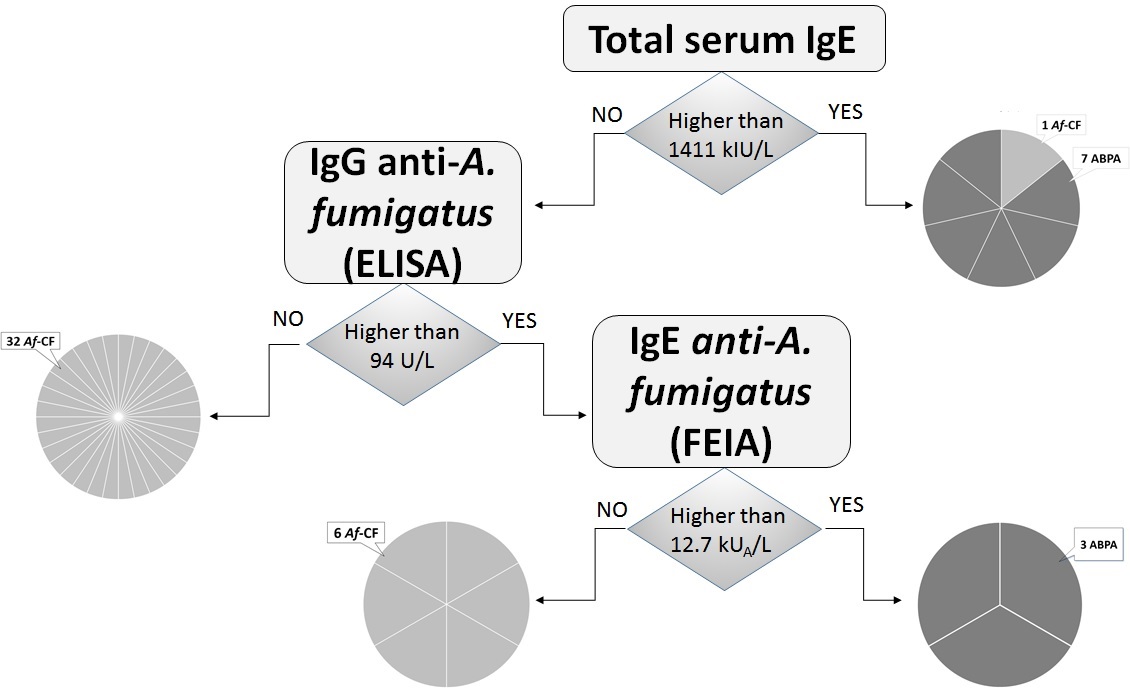

Supplement: Figure S1 — Classification tree using the classical laboratory measures comprised in the current diagnostic score of allergic bronchopulmonary aspergillosis (ABPA) (redrawn). The diagnostic algorithm retains total IgE, ELISA IgG, and specific IgE, yielding 2 Aspergillus fumigatus (Af)-cystic fibrosis (CF)-only groups counting 38/39 patients, 1 ABPA-only group of 3 patients, and 1 mixed group of 8 patients with an 88% probability of having ABPA. Overall, 38/39 Af-CF patients, but only 3/10 ABPA patients are clearly identified. [file Image_1.JPEG]

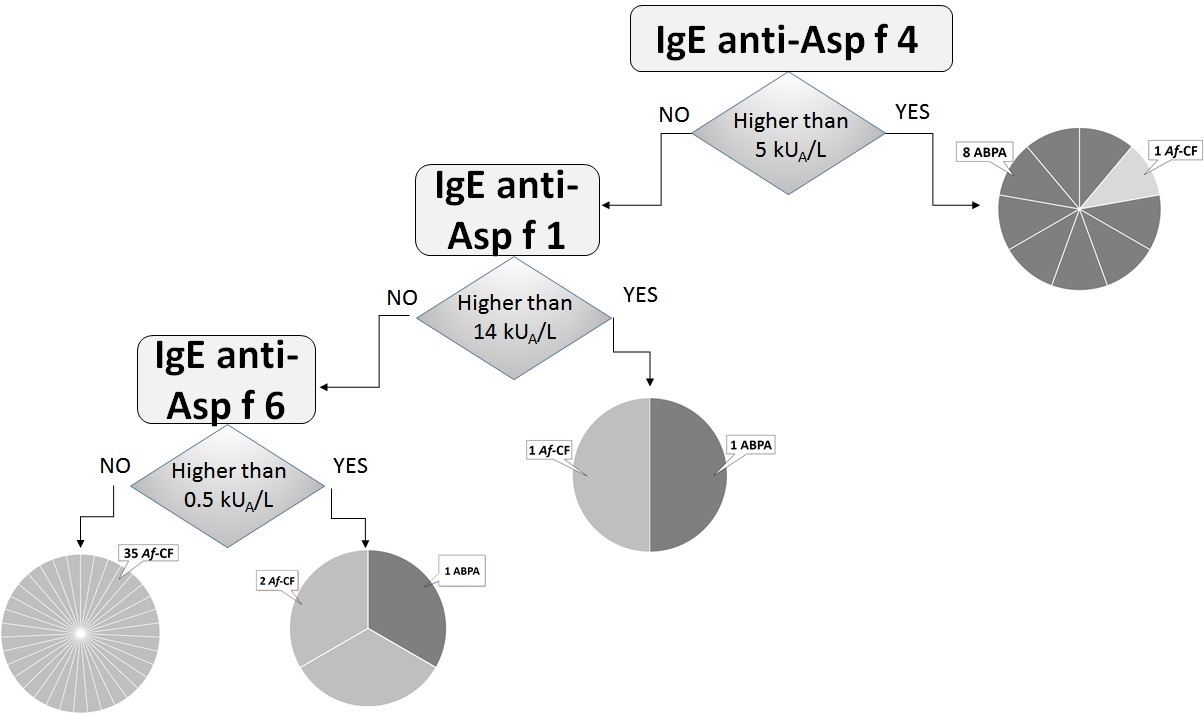

Supplement: Figure S2 — Classification tree analyzing sIgE responses to Asp f 1, Asp f 2, Asp f 3, Asp f 4, and Asp f 6 molecules. The resulting diagnostic algorithm retains IgE to Asp f 4, Asp f 1, and Asp f 6, yielding 1 Aspergillus fumigatus (Af)-cystic fibrosis (CF)-only group counting 35/39 patients, 1 mixed group of 9 patients with an 89% probability of having allergic bronchopulmonary aspergillosis (ABPA), and 2 mixed groups of undetermined clinical significance where other criteria are needed. Overall, 35/39 Af-CF patients, but no ABPA patients are clearly identified. [file Image_2.JPEG]
